# Identification of an Abbreviated Test Battery for Detection of HIV-Associated Neurocognitive Impairment in an Early-Managed HIV-Infected Cohort

**DOI:** 10.1371/journal.pone.0047310

**Published:** 2012-11-08

**Authors:** David J. Moore, Mollie J. P. Roediger, Lynn E. Eberly, Kaitlin Blackstone, Braden Hale, Amy Weintrob, Anuradha Ganesan, Brian K. Agan, Scott L. Letendre, Nancy F. Crum-Cianflone

**Affiliations:** 1 HIV Neurobehavioral Research Program, San Diego, California, United States of America; 2 Division of Biostatistics, University of Minnesota, Minneapolis, Minnesota, United States of America; 3 Infectious Disease Clinical Research Program, Uniformed Services University of the Health Sciences, Bethesda, Maryland, United States of America; 4 HIV Clinic, Naval Medical Center San Diego, San Diego, California, United States of America; 5 Naval Health Research Center, San Diego, California, United States of America; 6 Walter Reed National Military Medical Center, Bethesda, Maryland, United States of America; 7 Infectious Disease Clinic, National Naval Medical Center, Bethesda, Maryland, United States of America; Rush University, United States of America

## Abstract

**Background:**

HIV-associated neurocognitive disorders (HAND) remain prevalent despite improved antiretroviral treatment (ART), and it is essential to have a sensitive and specific HAND screening tool.

**Methods:**

Participants were 200 HIV-infected US military beneficiaries, managed early in the course of HIV infection, had few comorbidities, and had open access to ART. Participants completed a comprehensive, seven-domain (16-test), neuropsychological battery (∼120 min); neurocognitive impairment (NCI) was determined using a standardized score derived from demographically adjusted T-scores (global deficit score ≥0.5). Restricting the estimated administration time of the screening battery to < = 20 minutes, we examined the sensitivity and specificity of detecting NCI for all possible combinations of 2-, 3-, and 4- tests from the comprehensive battery.

**Results:**

Participants were relatively healthy (median CD4 count: 546 cells/mm^3^) with 64% receiving ART. Prevalence of NCI was low (19%). The best 2-test screener included the Stroop Color Test and the Hopkins Verbal Learning Test-Revised (11 min; sensitivity = 73%; specificity = 83%); the best 3-test screener included the above measures plus the Paced Auditory Serial Addition Test (PASAT; 16 min; sensitivity = 86%; specificity = 75%). The addition of Action Fluency to the above three tests improved specificity (18 min; sensitivity = 86%; specificity = 87%).

**Conclusions:**

Combinations of widely accepted neuropsychological tests with brief implementation time demonstrated good sensitivity and specificity compared to a time intensive neuropsychological test battery. Tests of verbal learning, attention/working memory, and processing speed are particularly useful in detecting NCI. Utilizing validated, easy to administer, traditional neuropsychological tests with established normative data may represent an excellent approach to screening for NCI in HIV.

## Introduction

HIV-associated neurocognitive disorders (HAND) remain prevalent despite improved antiretroviral treatment; up to 50% of HIV infected (HIV+) individuals are estimated to experience some level of neurocognitive impairment (NCI) [1]. The neurocognitive profile of HIV+ individuals is typically characterized by mild and “spotty” impairments; in fact, the most common form of HAND is “asymptomatic neurocognitive impairment” (ANI, estimated at 33% of the HIV+ population) [1], in which individuals experience impairment that does not reportedly interfere with their daily functioning. Original recommendations for the neurocognitive assessment of individuals with HIV from the National Institute of Mental Health (NIMH)-sponsored “AIDS Workshop: Neuropsychological Assessment Approaches” included an extensive (7–9 hr) and “brief” (1–2 hr) neuropsychological battery [2]. However, there is a growing demand for even briefer neurocognitive assessments, such as neurocognitive screening instruments, which can aid in the preliminary identification of individuals who may be appropriate to undergo further testing. These screening batteries or instruments would ensure an efficient use of time and resources in both clinical and research protocols [3]. However, parsimonious selection of assessment tools that are the most sensitive and specific to the mild NCI observed in HIV+ individuals has proven challenging.

Common traditional cognitive screening measures such as the Mini Mental Status Exam (MMSE) [3] and the Mattis Dementia Rating Scale (DRS) [4] were largely developed for dementing disorders and therefore primarily target cognitive functions (e.g., naming errors, gross visuospatial deficits) that are impaired as a result of posterior neocortical pathology. HIV-associated NCI, however, is typically milder in degree of impairment and more often involves pathophysiology within the fronto-striatal regions (e.g., processing speed) [5]–[7] than cortical dementias such as Alzheimer disease. As such, traditional cognitive screening measures are not typically sensitive enough for detecting HIV-related NCI [8], [9].

Due to the lack of sensitivity of traditional cognitive screeners, there have been attempts to develop screening instruments specific to persons with HIV disease. The HIV Dementia Scale (HDS) was developed to address these issues and has received widespread attention. Although the HDS has been shown to be more sensitive to the most severe form of HIV-related neurocognitive dysfunction (i.e., HIV-associated Dementia (HAD) [8]) than the traditional screeners (i.e., MMSE), it has not been able to reliably detect the more common form of mild HIV-related NCI [10]–[12]. For example, Morgan et al. [13] reported that even after demographically adjusting the scores on the HDS, the measure was still only 50% sensitive in detecting incidents of asymptomatic neurocognitive impairment.

Other neurocognitive screeners that have been examined in the context of HIV infection have also failed to show sufficient sensitivity to the mild NCI observed in the early stages of infection. For example, the Mental Alteration Test showed sensitivity to HIV-associated impairment comparable to the MMSE [14]; however, since prior studies suggest that the MMSE lacks sensitivity to mild HIV impairment [8], [9], it is unclear how useful the Mental Alteration Test is at detecting such deficits. Additionally, the Executive Interview (EXIT) was shown to be less sensitive to HIV-associated impairment than the HDS [15]. Lastly, Cogstate is a computerized neurocognitive screener which showed good sensitivity and specificity in individuals with advanced HIV disease (mean CD4 = 339, nadir CD4 = 76) and AIDS-defining complex (mean CD4 = 406, nadir CD4 = 137) [16], but has not been examined in less severely impaired HIV infected individuals. The International HDS (IHDS) was also developed to detect HIV-related dementia across global populations; however, for the purposes of the current study, this measure suffers from similar shortcomings as the original HDS in that it is designed to detect dementia, rather than the more mild forms of cognitive difficulties in HIV. Currently, the IHDS has been shown to be approximately 80% sensitive and 50% specific in detecting dementia among HIV+ U.S. and Ugandan individuals [17]. Therefore, the IHDS has limitations for detecting the more common mild cognitive difficulties experienced by individuals with HIV.

Also of importance when developing a novel neurocognitive screener is the consideration of the psychometric properties and ease of interpretation and clinical integration of a measure. When any novel measure is created, it is essential to establish that the validity and reliability of the measure are within the accepted range, a step that can be time-consuming and is often overlooked (e.g., reliability is not reported on the HDS). Instead, utilization of established neuropsychological measures as a cognitive screener may overcome some of these difficulties since these instruments have established psychometric properties and are widely utilized and recognized in the field of neuropsychology, which not only bolsters the validity and reliability of the screener, but also facilitates common interpretation of its results. For instance, Carey et al. [10] developed a screening battery to detect HIV-related NCI utilizing a two-measure combination of *a priori* selected traditional neuropsychological tests; this approach increases interpretability and familiarity of the screener to the clinician or researcher. Although the authors found good sensitivity (75–78%) by using an *a priori* selection of screening tests, it is not clear if the optimal measures, number of measures (e.g., the authors only included single and two test combinations), or the combination of measures was examined.

Therefore, in our study we aimed to identify the best combinations of traditional neuropsychological measures to be used as a screener, that show high sensitivity and specificity to a larger comprehensive neuropsychological battery by exploring all possible combinations of two, three, and four measures. By applying this approach to a cohort of early-managed HIV-infected patients, the best screener will reflect those measures that are most sensitive and specific to mild HIV-related NCI in a cohort with relatively low levels of overall impairment. Therefore, our goal is to present a menu of the most optimal measure combinations, where the definition of ‘optimal’ may be informed by sensitivity and specificity, time constraints, and/or feasibility (e.g., availability of measure materials, training).

## Methods

Participants were 200 HIV-infected US military beneficiaries, who were diagnosed early in the course of HIV infection, had few comorbidities, and had open access to antiretroviral medications. All participants were military beneficiaries with enrollment characteristics as previously described [18]. All study participants provided written informed consent and the study was approved by a central military institutional review board. The trial was registered at ClinicalTrials.gov (Registration #NCT00893815).

Participants completed a comprehensive, seven-domain (16-tests; 18 variables examined), neuropsychological battery (120 min) (Table 1). These tests cover a broad range of neuropsychological abilities and the battery is based on the one utilized by the large multi-site CHARTER study [1]. Raw neuropsychological (NP) test scores were converted into *T*-scores using demographically adjusted norms to control for the effects of age, education, gender, and where available ethnicity [19]–[21]. The demographically corrected *T-*scores were then converted into global (GDS) and domain deficit (DDS) scores according to a standardized approach [10], [22]. A GDS score greater than or equal to 0.5 was used to define global NP impairment.

**Table 1:** Component tests from neuropsychological battery, abbreviations, estimated administration time, and mean (SD) *T*-score performance for each of the measures in our sample of 200 HIV-infected individuals.

Test	Abbreviation	Admin Time (min)	*T*-score Mean (SD)	*Materials*	*Approximate Cost*	*Public domain vs. Copyrighted*
**Verbal Fluency**						
Letter Fluency	FAS	6	49.87 (9.66)	Paper score sheet, stopwatch	$0	Public domain
Animal Fluency	ANIMF	2	49.54 (9.54)	Paper score sheet, stopwatch	$0	Public domain
Action Fluency	ACTF	2	48.07 (10.32)	Paper score sheet, stopwatch	$0	Public domain
**Abstraction/Executive Functioning**						
Wisconsin Card Sorting Test (WCST) (Total Errors)	WCST	15	53.18 (11.50)	Computer program OR stimulus cards+paper score sheet	$820.00 (computer version) OR $375.00 (paper version)	Copyrighted
Trail Making Test - B	TMTB	5	50.51 (10.51)	Paper stimuli, pencil, stopwatch	$0	Public domain
Stroop Incongruent Test	STRPINC	1	49.98 (10.87)	Paper stimuli, stopwatch	$40.00–$149.00 (Stroop kit)	Copyrighted
**Speed of Information Processing**						
WAIS-III Symbol Search	SYMS	3	54.53 (9.47)	Paper stimuli, score sheet, pencil, stopwatch	$415.00 (WAIS-III kit)	Copyrighted
WAIS-III Digit Symbol	DSYM	3	51.15 (9.77)	Paper stimuli, score sheet, pencil, stopwatch	$415.00 (WAIS-III kit)	Copyrighted
Trail Making Test - A	TMTA	1	48.64 (9.65)	Paper stimuli, pencil, stopwatch	$0	Public domain
Stroop Color Test	STRPCOL	1	49.79 (11.53)	Paper stimuli, stopwatch	$40.00–$149.00 (Stroop kit)	Copyrighted
**Attention/Working Memory**						
Paced Auditory Serial Addition Test - 50	PASAT	5	46.10 (10.06)	Audio recording, paper score sheet	$0	Public domain
WAIS-III Digit Span	DSPAN	5	49.82 (9.65)	Paper score sheet	$415.00 (WAIS-III kit)	Copyrighted
**Learning**						
Brief Visuospatial Memory Test - Revised (Learning Trials)	BVMTR-LRN	10	51.82 (8.98)	Stimulus book, blank paper, pencil	$336.00 (BVMT-R kit)	Copyrighted
Hopkins Verbal Learning Test – Revised (Learning Trials)	HVLTR-LRN	10	44.09 (9.50)	Paper score sheet	$326.00 (HVLT-R kit)	Copyrighted
**Recall**						
Brief Visuospatial Memory Test - Revised (Total Recall)	BVMTR-RCL	30	50.38 (11.11)	Blank paper, pencil	$336.00 (BVMT-R kit)	Copyrighted
Hopkins Verbal Learning Test – Revised (Total Recall)	HVLTR-RCL	30	45.80 (9.57)	Paper score sheet	$326.00 (HVLT-R kit)	Copyrighted
**Motor Speed and Dexterity**						
Grooved Pegboard - dominant hand	PD	3	45.05 (9.20)	Pegboard, pegs, stopwatch	$73–$218	Copyrighted
Grooved Pegboard - non-dominant hand	PND	3	44.11 (9.52)	Pegboard, pegs, stopwatch	$73–$218	Copyrighted

Our goal was to establish a screening battery that was brief to administer and was both sensitive and specific to NCI as determined by global NP impairment, described above. All possible combinations of 2-, 3-, and 4- NP tests from the comprehensive battery, limited to those combinations that took no more than 20 minutes to administer, were considered (The time limitation necessarily excluded the Hopkins Verbal Learning Test-Revised and the Brief Visuospatial Memory Test-Revised Delayed Recall measures from any possible combination). To maximize domain breadth assessed by the combinations all possible combinations, regardless of domain, were considered; the approach yielded the following potential screening battery combinations: 153 2-test, 816 3-test, and 3060 4-test combinations. For each 2-test combination, screening NCI was defined as either two tests with *T*-score<40 or one test with *T*-score<35. For each 3-test combination, screening NCI was defined as meeting one of the following three criteria: (1) three tests with *T*-score<40; (2) one test with *T*-score<40 and one test with *T*-score<35; or (3) one test with *T*-score<30. For each 4-test combination, screening NCI was defined as meeting one of the following five criteria: (1) four tests with *T*-score<40; (2) two tests with *T*-score<40 and one test with *T*-score<35; (3) two tests with *T*-score<35; (4) one test with *T*-score<40 and one test with *T*-score<30; or (5) one test with *T*-score<25. The rationale for this scoring structure was that these combinations would always generate a screening GDS equivalent to 1.0, and is similar to a previously published technique [10]. This screening level for impairment is double that required for impairment with our comprehensive battery (i.e., GDS = 0.05). A more substantial level of impairment is required for the screening tests in order to provide a conservative approach to identifying persons who might be at risk for neurocognitive impairment with a larger overall battery.

Sensitivity, specificity, negative predictive value (NPV), positive predictive value (PPV), and odds ratios (OR) were calculated separately for each screening test combination using the global NP impairment from the comprehensive battery as the “gold standard”. The testing combinations were then ranked by the sum of their sensitivity and specificity. To assure that the classification accuracy estimates were not sensitive to sample anomalies, the data were randomly re-sampled 10,000 times, with replacement (bootstrapped). The bias-corrected and accelerated (BCa) interval method [23] was used to produce 95% confidence intervals around the estimates for sensitivity, specificity, NPV, and PPV. The listings of possible combinations of test from independent domains were generated using perl scripts; statistical analyses were conducted using SAS software [24].

## Results

Our HIV-infected cohort was predominantly male (95.5%), relatively young (median age 36.4 years, interquartile range (IQR) 28.1–43.6), racially and ethnically diverse (48.5% White, 29% Black, 14.0% Hispanic, and 8.5% other race/ethnicity), well educated (34% with post-secondary degree), had few comorbidities (only seven reported current illicit drug use and three had hepatitis C), and healthy (median CD4 count: 546 cells/mm3, IQR 417–706) with 64% receiving ART. The median duration of HIV infection was 5 years (IQR 2–11 years). Prevalence of global NP impairment was low (19%).

Rather than decide the optimal combination of tests to define one specific screening battery, we have chosen to present an enumeration of the top-ranked combinations; this provides users with the information needed to select the highest-ranked combination from among those that satisfy site constraints in time, training, cost, or equipment (Table 1).

Tables 2, 3, and 4 provide the test combinations that are the ten highest-ranked for 2, 3, and 4-test combinations, respectively. The 2-test screening combination with the best sensitivity and specificity for global NP impairment included the Stroop Color Test and the Hopkins Verbal Learning Test-Revised Learning Trials (11 minutes; sensitivity = 73%; specificity = 83%; Table 2). Interestingly, all of the ten best 2-test combinations included the Stroop Color Test (measuring processing speed), the PASAT (measuring working memory), or the Hopkins Verbal Learning Test-Revised Learning Trials (measuring learning), and the highest-ranked 3-test combination included exactly these three measures (16 minutes; sensitivity = 86%; specificity = 75%; Table 3). The optimal 4-test screening battery added the Action Fluency test to the same tests found in the best 3-test combination and improved specificity but not sensitivity (18 minutes; sensitivity = 86%; specificity = 87%; Table 4).

**Table 2:** Top 10 2-test combinations, ranked according to sum of sensitivity and specificity compared to a 120 minute global neuropsychological battery.

Rank	Tests	Administration Time (min)	Sensitivity (95% CI)^1^	Specificity (95% CI)^1^	PPV (95% CI)^1^	NPV (95% CI)^1^	OR (95% CI)
1	STRPCOL/HVLTR-LRN	11	73.0 (55.6–85.7)	83.1 (76.3–88.2)	50.0 (35.0–62.1)	93.0 (87.7–96.5)	13.3 (5.8–30.7)
2	HVLTR-LRN/PD	13	73.7 (56.7–86.1)	82.0 (75.0–87.1)	49.1 (35.4–61.7)	93.0 (87.5–96.4)	12.7 (5.6–29.1)
3	PASAT/BVMTR-LRN	15	63.2 (46.3–77.8)	89.7 (84.3–93.8)	60.0 (43.9–74.3)	90.9 (85.4–94.8)	15.0 (6.5–34.7)
4	PASAT/HVLTR-LRN	15	73.7 (56.8–86.2)	77.6 (70.4–83.3)	44.4 (31.7–56.5)	92.4 (86.6–96.2)	9.7 (4.3–21.9)
5	PASAT/PND	8	71.1 (54.3–84.2)	79.5 (72.6–85.4)	45.8 (32.8–58.8)	91.9 (86.0–95.6)	9.5 (4.3–21.2)
6	HVLTR-LRN/PND	13	71.1 (54.1–83.9)	77.6 (70.4–83.3)	42.9 (30.0–55.1)	91.9 (86.2–95.7)	8.5 (3.9–18.8)
7	STRPCOL/BVMTR-LRN	11	54.1 (37.1–69.7)	94.4 (89.7–97.4)	69.0 (50.0–84.2)	89.9 (84.5–93.9)	19.7 (7.8–50.2)
8	STRPCOL/PD	4	56.8 (40.0–71.8)	90.6 (85.2–94.4)	58.3 (40.7–73.7)	90.1 (84.5–94.0)	12.7 (5.5–29.4)
9	STRPINC/HVLTR-LRN	11	64.9 (47.2–79.3)	82.5 (75.4–87.6)	46.2 (31.8–59.6)	91.0 (85.4–95.0)	8.7 (4.0–19.1)
10	PASAT/WCST	20	60.0 (41.7–75.0)	86.9 (81.0–91.7)	51.2 (35.3–66.7)	90.5 (84.8–94.6)	10.0 (4.4–22.7)

1 95% bootstrap CI for Sensitivity, Specificity, PPV and NPV.

**Abbreviations:** BVMTR-LRN, Brief Visuospatial Memory Test-Revised (Learning Trials); CI, Confidence Interval; HVLTR-LRN, Hopkins Verbal Learning Test-Revised (Learning Trials); NPV, Negative Predictive Value; OR, Odds Ratio; PASAT, Paced Auditory Serial Addition Test; PD, Grooved Pegboard-Dominant hand; PND, Grooved Pegboard-non Dominant hand; PPV, Positive Predictive Value; STRPCOL, Stroop Color Test; STRPINC, Stroop Incongruent Test; WCST, Wisconsin Card Sorting Test (Total Errors).

**Table 3:** Top 10 3-test combinations, ranked according to sum of sensitivity and specificity compared to a 120 minute global neuropsychological battery.

Rank	Tests	Admin. Time (min)	Sensitivity (95% CI)^1^	Specificity (95% CI)^1^	PPV (95% CI)^1^	NPV (95% CI)^1^	OR (95% CI)
1	STRPCOL/PASAT/HVLTR-LRN	16	86.5 (71.4–95.1)	75.5 (67.9–81.6)	45.7 (33.8–57.0)	95.9 (90.7–98.4)	19.7 (7.2–54.2)
2	TMTA/PASAT/HVLTR-LRN	16	84.2 (69.4–93.9)	76.3 (69.0–82.2)	46.4 (33.9–58.0)	95.2 (90.4–98.3)	17.2 (6.7–44.2)
3	SYMS/PASAT/HVLTR-LRN	18	78.9 (62.8–90.3)	79.5 (72.4–85.0)	48.4 (35.2–60.3)	93.9 (88.5–97.1)	14.5 (6.1–34.7)
4	ACTF/STRPCOL/HVLTR-LRN	13	78.4 (61.9–89.7)	80.0 (72.6–85.4)	47.5 (34.4–59.7)	94.1 (89.0–97.2)	14.5 (6.1–34.7)
5	PASAT/BVMTR-LRN/PND	18	76.3 (60.0–88.2)	82.1 (75.6–87.7)	50.9 (37.8–64.1)	93.4 (88.0–96.9)	14.7 (6.3–34.5)
6	PASAT/HVLTR-LRN/PD	18	81.6 (65.5–91.9)	75.6 (68.1–81.6)	44.9 (32.9–56.3)	94.4 (89.1–97.6)	13.8 (5.6–33.8)
7	STRPCOL/HVLTR-LRN/PD	14	78.4 (61.8–89.7)	78.8 (71.4–84.5)	46.0 (33.3–58.1)	94.0 (88.7–97.1)	13.4 (5.6–32.1)
8	TMTA/PASAT/PND	9	76.3 (60.0–88.2)	80.1 (73.5–85.9)	48.3 (35.5–61.3)	93.3 (87.8–96.8)	13.0 (5.6–30.2)
9	ANIMF/STRPCOL/HVLTR-LRN	13	75.7 (59.1–87.5)	80.6 (73.6–86.0)	47.5 (34.0–60.0)	93.5 (88.0–96.9)	12.9 (5.5–30.2)
10	FAS/STRPCOL/BVMTR-LRN	17	64.9 (47.4–78.8)	91.3 (85.9–95.1)	63.2 (46.2–77.8)	91.8 (86.6–95.5)	19.3 (8.1–45.9)

1 95% bootstrap CI for Sensitivity, Specificity, PPV and NPV.

**Abbreviations:** ACTF, Action Fluency; ANIMF, Animal Fluency; BVMTR-LRN, Brief Visuospatial Memory Test-Revised (Learning Trials); CI, Confidence Interval; FAS; Letter Fluency; HVLTR-LRN, Hopkins Verbal Learning Test-Revised (Learning Trials); NPV, Negative Predictive Value; OR, Odds Ratio; PASAT, Paced Auditory Serial Addition Test; PD, Grooved Pegboard-Dominant hand; PND, Grooved Pegboard -non Dominant hand; PPV, Positive Predictive Value; STRPCOL, Stroop Color Test; SYMS, WAIS-III Symbol Search; TMTA, Trail Making Test-A.

**Table 4:** Top 10 4-test combinations, ranked according to sum of sensitivity and specificity compared to a 120 minute global neuropsychological battery.

Rank	Tests	Admin. Time (min)	Sensitivity (95% CI)^1^	Specificity (95% CI)^1^	PPV (95% CI)^1^	NPV (95% CI)^1^	OR (95% CI)
1	ACTF/STRPCOL/PASAT/HVLTR-LRN	18	86.5 (71.1–95.0)	87.1 (80.8–91.7)	61.5 (47.1–74.1)	96.4 (92.0–98.6)	43.2 (15.1–124)
2	STRPCOL/PASAT/HVLTR-LRN/PND	19	83.8 (68.3–93.8)	83.2 (76.7–88.7)	54.4 (41.0–67.2)	95.6 (90.6–98.4)	25.6 (9.7–67.7)
3	STRPCOL/PASAT/HVLTR-LRN/PD	19	81.1 (65.1–91.7)	85.2 (78.8–90.2)	56.6 (42.6–69.2)	95.0 (90.1–97.8)	24.6 (9.7–62.6)
4	ACTF/STRPCOL/PASAT/BVMTR-LRN	18	73.0 (56.3–85.7)	92.9 (87.7–96.2)	71.1 (54.1–83.8)	93.5 (88.5–96.7)	35.3 (13.7–91.4)
5	ACTF/TMTA/PASAT/HVLTR-LRN	18	81.6 (65.9–91.9)	84.0 (77.6–89.1)	55.4 (41.5–67.9)	94.9 (90.1–97.8)	23.2 (9.2–58.5)
6	ANIMF/STRPCOL/PASAT/HVLTR-LRN	18	78.4 (62.2–89.5)	86.5 (80.4–91.3)	58.0 (43.4–71.1)	94.4 (89.3–97.3)	23.1 (9.3–57.3)
7	FAS/STRPCOL/PASAT/PND	15	75.7 (59.0–87.8)	88.4 (82.4–92.8)	60.9 (45.2–74.3)	93.8 (88.7–97.1)	23.7 (9.7–58.1)
8	ACTF/STRPCOL/PASAT/PND	11	75.7 (58.5–87.5)	87.7 (81.6–92.3)	59.6 (44.2–73.0)	93.8 (88.6–97.1)	22.3 (9.1–54.3)
9	ACTF/SYMS/PASAT/HVLTR-LRN	20	73.7 (57.1–86.1)	88.5 (82.4–92.8)	60.9 (45.5–74.1)	93.2 (88.2–96.6)	21.5 (9.0–51.4)
10	ACTF/DSYM/PASAT/HVLTR-LRN	20	73.7 (57.1–86.1)	88.2 (82.1–92.6)	60.9 (45.5–74.1)	93.1 (87.8–96.5)	21.0 (8.8–50.3)

1 95% bootstrap CI for Sensitivity, Specificity, PPV and NPV.

**Abbreviations:** ACTF, Action Fluency; ANIMF, Animal Fluency; BVMTR-LRN, Brief Visuospatial Memory Test-Revised (Learning Trials); CI, Confidence Interval; DSYM, WAIS-III Digit Symbol; FAS, Letter Fluency; HVLTR-LRN, Hopkins Verbal Learning Test-Revised (Learning Trials); NPV, Negative Predictive Value; OR, Odds Ratio; PASAT, Paced Auditory Serial Addition Test; PD, Grooved Pegboard-Dominant hand; PND, Grooved Pegboard -non Dominant hand; PPV, Positive Predictive Value; STRPCOL, Stroop Color Test; SYMS, WAIS-III Symbol Search; TMTA, Trail Making Test-A.

All of the ten best 3-test combinations showed better summed sensitivity and specificity than the best 2-test combination. Some 3-test combinations required comparable or even less administration time and showed equivalent sensitivity plus specificity as the best 2-test combination (Tables 2–3). For example, a screening combination consisting of the Trail Making Test Part A, PASAT and Grooved Pegboard (Non-dominant hand) takes a total of 9 minutes to administer and had slightly higher sensitivity (76%) plus specificity than the best 2-test combination which takes 11 minutes to administer. Several other combinations of very brief screening (<10 min) combinations yielded good sensitivity and specificity as well (Table 5).

**Table 5:** Top 10 testing combinations, ranked according to sum of sensitivity and specificity compared to a 120 minute global neuropsychological battery, that take 10 minutes or less to administer.

Rank	Tests	Admin. Time (min)	Sensitivity (95% CI)^1^	Specificity (95% CI)^1^	PPV (95% CI)^1^	NPV (95% CI)^1^	OR (95% CI)
1	TMTA/PASAT/PND	9	76.3 (60.0–88.2)	80.1 (73.5–85.9)	48.3 (35.5–61.3)	93.3 (87.8–96.8)	13.0 (5.6–30.2)
2	ACTF/STRPCOL/PD/PND	9	70.3 (53.3–83.3)	88.1 (82.5–92.6)	57.8 (42.6–72.0)	92.8 (87.7–96.2)	17.5 (7.5–41.1)
3	STRPCOL/PASAT/PND	9	75.7 (58.6–87.8)	78.1 (71.2–84.2)	45.2 (32.8–58.2)	93.1 (87.4–96.7)	11.1 (4.8–25.7)
4	ACTF/STRPCOL/DSYM/PD	9	59.5 (42.1–74.5)	94.3 (89.4–97.3)	71.0 (52.0–85.2)	90.8 (85.4–94.6)	24.1 (9.4–61.7)
5	ACTF/STRPCOL/TMTA/PND	7	62.2 (45.0–76.9)	91.3 (86.0–94.9)	62.2 (45.2–76.9)	91.3 (86.0–95.0)	17.1 (7.2–40.5)
6	STRPCOL/STRPINC/PASAT/PND	10	67.6 (50.0–81.5)	85.8 (79.5–90.7)	53.2 (38.1–67.4)	91.7 (86.2–95.6)	12.6 (5.5–28.7)
7	STRPCOL/TMTA/PASAT/PND	10	67.6 (50.0–81.6)	85.8 (79.7–90.7)	53.2 (38.1–67.4)	91.7 (86.2–95.6)	12.6 (5.5–28.7)
8	ACTF/PASAT/PND	10	76.3 (59.5–88.1)	76.9 (69.9–83.1)	44.6 (32.4–56.9)	93.0 (87.3–96.7)	10.7 (4.7–24.8)
9	ACTF/STRPCOL/PND	6	70.3 (54.1–83.8)	82.5 (76.0–87.9)	48.1 (34.5–62.1)	92.3 (86.9–95.9)	11.1 (4.9–25.2)
10	STRPINC/TMTA/PASAT/PND	10	67.6 (50.0–81.6)	85.2 (78.7–90.2)	52.1 (37.3–66.7)	91.7 (86.2–95.5)	12.0 (5.3–27.1)

1 95% bootstrap CI for Sensitivity, Specificity, PPV and NPV.

**Abbreviations:** ACTF, Action Fluency; CI, Confidence Interval; FAS, Letter Fluency; NPV, Negative Predictive Value; OR, Odds Ratio; PASAT, Paced Auditory Serial Addition Test; PD, Grooved Pegboard-Dominant hand; PND, Grooved Pegboard -non Dominant hand; PPV, Positive Predictive Value; STRPCOL, Stroop Color Test; STROOPINC, Stroop Incongruent Test; TMTA, Trail Making Test-A.

In examining our results with an eye toward practicality, we considered combinations of measures that would not require specialized equipment (e.g., the PASAT 50 requires an audio player, Grooved Pegboard Test requires a pegboard). The most sensitive and specific combination of tests that could be administered with limited testing stimuli (i.e., examiner, paper testing stimuli, and pencil) was Action Fluency, Stroop Color Test, Trail Making Test – B, and the Hopkins Verbal Learning Test-Revised Learning Trials (18 minutes; sensitivity = 70.3%; specificity = 89.4%). Although all neuropsychological tests require a trained examiner for reliable administration, any of the top combinations of tests require relatively minimal training to administer (e.g., mostly the reading of directions and the recording of responses by the examiner); an examiner could be trained to administer and score any particular combination of measures in a limited amount of time (i.e., less than 2 hours of training).

In order to further validate the reported test combinations, we also analyzed and compared a previously published combination of tests that has been used in individuals with HIV [25] in our cohort. This 3-test combination, which included Trail Making Test – A and B and Digit Symbol, showed poor sensitivity (36.8%) but good specificity (92.5%) in our study cohort of mildly impaired HIV individuals.

## Discussion

Our study reveals that several combinations of traditional neuropsychological tests that require relatively little administration time (i.e., <20 minutes, and in some cases <10 minutes) can yield good sensitivity and specificity in identifying neurocognitive impairment as assessed by a larger test battery in a relatively high functioning sample of HIV-infected military beneficiaries. There is a great need to identify a sensitive and specific brief cognitive screening battery for detection of mild HIV-related neurocognitive impairment [26]. This is especially true given that cognition impacts medication adherence, employment and quality of life measures [20], [27]–[29]. Additionally, among service members, detection of neurocognitive impairment has critical occupational implications [27]. Tests that tended to be the most sensitive were in the domains of verbal learning, attention/working memory, and processing speed, which is generally consistent with the domains found to be impaired in larger studies of neurocognitive impairment in HIV [30]. These most sensitive and specific domains have been shown to be associated with the frontostriatal neural systems which are commonly disturbed among persons with HIV-infection (for a review see [31]).

Our approach, one that examined all possible test combinations across various neurocognitive domains, was empirically driven and unbiased by expectations of what combination of tests would likely be most sensitive and specific. We presented a menu of options, rather than advocate for a specific battery. While this may lead to inconsistent approaches across settings, we believed it was important to not mandate a “one size fits all” approach. If brevity is most important, the preferred choice appears to be Trail Making Part A, PASAT and the Grooved Pegboard non-dominant hand (9 minutes; 76% sensitivity). While special equipment is required for this battery, only the Grooved Pegboard ($110 at the time of submission of this manuscript) and a device to play an audio file (e.g., computer, CD player, or even a Smartphone) have an associated cost. The PASAT sound file and the Trail Making Part A are in the public domain. Thus, for a small initial investment and less than 10 minutes of administration time, one could screen for neurocognitive impairment utilizing reliable, well-validated measures. The screener with the best combination of sensitivity and specificity (i.e., Action Fluency, Stroop Color, PASAT, and HVLT-R Learning Trials) requires 7 more minutes of assessment time, but is also easy to administer. The two-minute investment to administer Action Fluency appears worthwhile for improved specificity with this battery (i.e., 87.1% versus 75.5%) as compared to the 3-test combination that does not include Action Fluency. The second ranked 3-test combination of Trail Making Test Part A, PASAT, and HVLT-R Learning avoids the requirement of having the color stimuli of the Stroop tests and replaces it with the Trail Making Test, which is widely available and in the public domain. If sensitivity were the most important criteria (which is likely given the desire to identify those who are abnormal), this battery, as well as the top ranked 3-test battery, would seem to be reasonable choices.

The menu of options is also important for multiple testing sessions over time. We know that a subset of HIV-infected persons can have fluctuations in cognitive ability over time and that neuropsychological tests are susceptible to practice effects [32]. One could consider switching to non-overlapping screening batteries at different assessment time points to avoid practice effect problems; however, this would require those who administer the tests to be trained on a wider range of instruments and for all assessment instruments to be available, which may not be feasible in some settings. In addition, multiple alternative forms are available for the HVLT-R (and BVMT-R), which is advantageous for eliminating practice effects on those tests that may be most susceptible to these problems (e.g., tests of learning/memory).

There are several advantages of using traditional neuropsychological tests as screening batteries as opposed to newly developed screening instruments. Specifically, all of the measures reported in this study have excellent normative data that allow for corrections for demographic factors that can influence neurocognitive test performance such as age, education, sex and ethnicity. All of the measures have been previously validated on large samples with excellent reliability and validity. Moreover, most of these tests are very easy to administer and interpret.

On the other hand, there are some disadvantages of using these tests. Some measures are copyrighted and have an associated cost as compared to a public domain screening instrument such as the Montreal Cognitive Assessment (MoCA) [33]. Also, in order to capitalize on the normative data, the raw scores would have to be converted into demographically corrected scores using a table look-up or a computerized program and there is an investment for these materials. One can argue, however, that utilizing a screener that does not account for certain demographic factors (in particular age, education, ethnicity) is inappropriate when these variables are known to affect cognitive functioning [20]. For a screening instrument, it may be best to avoid using tests that require some expertise in scoring (e.g., visual learning/memory tests) [34], and in this study other combinations of tests were more sensitive and specific.

For neuroAIDS research, there may be an advantage of the HIV field moving toward a consistent NP battery and standardized approaches to summarizing neurocognitive data. For example, some consensus approaches such as the MATRICS battery in schizophrenia have improved consistency across studies in that research arena (www.matrics.ucla.edu). One possible option for consistency moving forward may be the utilization of the NIH Tool Box (http://www.nihtoolbox.org). As of this writing, the specific battery of tests is not available, but the subdomains have been identified and beta testing of the battery is well underway. Studies will be needed to compare traditional neuropsychological assessment measures with those in the NIH Tool Box. With this said, in clinical settings, flexibility and brevity, with good sensitivity and specificity to larger neuropsychological assessment batteries are paramount; thus, the suggested screening combinations presented herein may have particular utility.

The present study is not without limitations. First, our overall impairment rate was very low when utilizing the comprehensive NP battery (19%); therefore, it is difficult to choose a subset of tests to screen for impairment when impairment is relatively limited. The present screening battery may be most appropriate for well-treated HIV populations with few comorbidities, and ideally, participants identified with impairment would be referred for additional more detailed neurocogntive assessment. Another limitation is the choice to include the same NP measures in both the screening battery and the comprehensive NP battery. We believed that eliminating these measures and recalculating a score from the larger battery without these measures would lead to less stable characterization of the cohort across combination examinations. In order to further address the lack of a gold standard in the current study, however, we calculated and compared a previously published 3-test combination (i.e., Trail Making Test – A and B and Digit Symbol) [25] within our study cohort. We found that this established 3-test battery was not as sensitive (i.e., 36.8%) as other test combinations that were identified with our methods, which renders further support for the combinations identified in our study. Due to these limitations, our screening batteries need validation among other HIV-infected populations. Lastly, although our screening batteries may show good sensitivity in detecting neurocognitive impairment, it is important to note that the batteries are not necessarily diagnostic of HIV-associated neurocognitive impairment since other potential causes of neurocognitive impairment must be ruled out before the neurocognitive impairment can be deemed to be due to HIV. Moreover, our screening batteries are not meant to diagnose HIV-associated neurocognitive disorders (HAND) given that a HAND diagnosis additionally requires a determination of daily functioning ability [32].

In summary, combinations of widely accepted neuropsychological tests with short implementation times demonstrated adequate sensitivity and specificity compared to a more time intensive NP test battery. Tests of verbal learning, attention/working memory, and processing speed appeared to be particularly useful in detecting NCI. While several screening instruments have been developed for the detection of HIV-associated NCI, utilizing a combination of validated, relatively easy to administer, neuropsychological tests with established normative data may represent an excellent approach to detecting NCI.
